# When Care Faces Violence: Anticipatory Grief, Chronic Vigilance, and Ambiguous Loss Among Street Dog Care-Givers in Istanbul

**DOI:** 10.3390/ani16040559

**Published:** 2026-02-11

**Authors:** Mine Yıldırım

**Affiliations:** Department of Common Courses, Kadir Has University, Cibali, 34083 Istanbul, Turkey; mine.yildirim@khas.edu.tr

**Keywords:** street dogs, animal caregivers, caregiver well-being, anticipatory grief, chronic vigilance, ambiguous loss, veterinary access, animal welfare governance, Istanbul

## Abstract

This article examines how Turkey’s 2024 amendment to the Animal Protection Law reshapes volunteer caregiving for free-roaming dogs in Istanbul by reconfiguring the practical conditions under which care is sought, coordinated, and sustained. Drawing on 43 in-depth interviews and five months of fieldwork (1 July–30 November 2025), this study combines constructivist grounded theory with reflexive thematic analysis to trace how legal change is encountered through everyday governance interfaces and how these encounters reorganize caregivers’ routines, capacities, and moral worlds. The analysis yields four interlocking findings. First, caregivers describe a temporality of “living in pre-loss,” in which anticipated removal, disappearance, and uncertain outcomes generate chronic vigilance, anticipatory grief, and ambiguous loss without closure. Second, caregiving is increasingly recalibrated as risk management: commitments persist, but intervention narrows through heightened exposure to complaints, reputational scrutiny, and fears that help-seeking may backfire. Third, institutional pathways—hotlines, shelter intake, and municipal responses—are experienced as discretionary and opaque, producing a fluctuating threshold between assistance and harm that conditions whether caregivers engage official systems at all. Fourth, this study identifies a recurring veterinary bottleneck at the street–clinic–recovery handover, where limited short-term holding capacity stalls treatment trajectories and displaces recovery labor into precarious domestic and informal spaces. Together, these findings argue that caregiver well-being is not ancillary to animal welfare governance but constitutive of it. It shapes the continuity of monitoring, the timeliness of intervention, and the everyday mediation through which coexistence is maintained under intensified legal and political pressure.

## Simple Summary

Many studies of human–animal relationships focus on pets in the home, but in Istanbul thousands of bonds are formed and sustained in public space through unpaid community caregiving. This qualitative study examines how volunteers who care for free-roaming street dogs experience daily life after Turkey’s 2024 amendment to the Animal Protection Law, which intensified pressure to remove dogs from streets. Based on 43 in-depth interviews with caregivers and five months of fieldwork, the article shows how care becomes shaped by uncertainty about municipal responses and the risk that seeking help can lead to capture or confinement. Caregivers describe living with constant vigilance, anticipatory grief, and ambiguous loss when dogs disappear or are taken without confirmation. These pressures often shift caregiving from open-ended commitment toward ongoing risk management. The study also identifies a recurring welfare bottleneck: many private clinics can treat street dogs but cannot provide short-term holding for recovery, forcing caregivers to improvise temporary spaces and turning treatable cases into emergencies. Overall, the findings show that caregiver well-being, institutional design, and veterinary capacity are tightly linked to urban animal welfare and the possibility of humane coexistence beyond the household.

## 1. Introduction

Across towns and cities in Turkey, the everyday survival of street dogs is sustained by dispersed volunteer labor and care work. Caregivers feed and monitor dogs, arrange ad hoc veterinary care, mediate neighborhood tensions, and respond to crises. These routine practices constitute an informal care infrastructure: when it holds, minor injuries are treated before they escalate, frictions are defused before they become formal complaints, and dogs are more likely to remain visible, tolerated, and alive. When it frays, avoidable suffering increases and relations of coexistence deteriorate.

This volunteer infrastructure does not operate in an institutional vacuum. Turkey’s formal system for “dealing with” street dogs is legally organized around municipal responsibility: capture and transport; “rehabilitation” (typically vaccination, spay/neutering, treatment, and identification); and the operation of municipal animal shelters (officially titled “rehabilitation centers”), with intake often triggered by complaints and governed by discretionary, street-level triage. The scale of this municipal system is substantial on paper yet strikingly limited relative to need. When parliament passed the 2024 reform, the bill’s own figures cited 322 shelters nationwide with a capacity of roughly 105,000 dogs, against state estimates in the millions—a gap that structurally preconditions reliance on households, volunteers, and NGOs for basic continuity of care [1]. Reporting on the reform highlights mandated earmarks for municipalities to build or upgrade shelters and deliver rehabilitation services, alongside an extended compliance horizon to 2028—precisely the kind of “deadline without capacity” dynamic that expands the practical burden placed on civil society [1,2,3,4,5].

Alongside municipal provision, a heterogeneous animal welfare politics operates through NGOs, rescue associations, local sheltering initiatives, and informal neighborhood networks that provide food distribution, spay/neutering support, emergency transport, fostering, and legal advocacy for street dogs. Yet these resources are unevenly distributed (concentrated in large metropolitan areas and a subset of coastal and touristic towns), chronically overstretched, and frequently forced into “patching” roles that bridge gaps between street, clinic, shelter, and recovery that the municipal system cannot reliably absorb. In this sense, volunteers are not peripheral to street-dog governance in Turkey. They function as a de facto extension of the system’s operational capacity and as its shock absorbers when official pathways are slow, opaque, or feared.

Any account of this governance landscape must begin with the uncomfortable fact that population size itself is politically contested. Public discussion and policy rationales commonly cite state estimates of around four million free-roaming stray dogs [6,7,8,9,10]. It is important to note that figures are often rounded up when discourse emphasizes dogs’ “strayness” and out-of-placeness. Other institutional and expert claims circulate figures that are substantially higher or lower, underscoring that the size of the street-dog population is not simply a demographic question but a battleground over legitimacy, urgency, and acceptable intervention [11]. More stable than any single number is the spatial patterning [12,13], which is deeply rooted in the history of modernization in Istanbul [14]. Free-roaming dogs are present across urban and rural Turkey, yet they are hyper-visible in dense metropolitan districts where food waste, commercial corridors, and constant human movement shape canine survival [15,16,17,18]. Variation across districts matters; Istanbul has long been studied through differentiated urban zones [19]. These differences are consequential because conflict between humans and dogs, and care for street dogs, do not distribute evenly. Instead, they cluster where dogs and humans are forced into repeated, intimate encounters over mobility, fear, and attitudes, mediated through the contested meanings and perceived legitimacy of public space [20,21,22].

The existing literature situates these dynamics as a long-standing—albeit periodically intensified—question of coexistence rather than a “new” social problem. Research on urban dogs and public space in Turkey has shown how street dogs are alternately framed as neighborhood cohabitants, infrastructures of affect, nuisances, and security threats, and how care is reconfigured as a privatized burden when the state governs through removal rather than sustained welfare provision [23]. Research focused on Istanbul further documents municipal capture practices (including displacement to peripheral spaces) and the biopolitics of community care under shifting legal regimes [19,24,25,26]. More recent analyses trace how the “stray dog problem” is discursively produced and contested in mediated publics, clarifying why everyday encounters so quickly become moralized conflicts over safety, classed respectability, and legitimate uses of the street [27,28,29].

The 2024 amendment to Turkey’s Animal Protection Law (Law No. 5199) sharpened the state’s shift toward removing free-roaming dogs from public space. In policy terms, the reform shifts the center of gravity away from the long-standing “capture–neuter–vaccinate–return” (CNVR) logic and toward large-scale collection and shelter-based management oriented toward adoption while also authorizing euthanasia under specified conditions (e.g., illness, pain, or perceived risk or aggression) [3,4]. Legal commentaries on the amendment similarly foreground tightened municipal obligations to collect dogs and keep them within “care shelter” pathways rather than returning them to where they were found, alongside strengthened restrictions and penalties aimed at preventing “release back to the street” [5]. A basic tension sits at the center of the reform: municipalities are tasked with rapid, large-scale removal despite a shelter system whose capacity represents only a small fraction of the estimated street-dog population. Parliamentary debate has cited an estimate of roughly four million free-roaming dogs nationwide while simultaneously acknowledging a shelter infrastructure consisting of only a few hundred facilities with total capacity far below that population [2,3,4,5,30]. Reporting has also documented continuing infrastructural strain and uneven municipal readiness, including concerns about shelter conditions and capacity limits [31]. The reform thus intensifies removal expectations while leaving this capacity gap largely unresolved [32].

The political and public rationale for this shift has been built around highly publicized dog bites, “rabies-suspected injuries”, and death cases, alongside an intensified “safe streets” discourse and recurring claims that the previous legal framework was insufficient to protect public health and safety [3,33,34,35,36]. Media coverage of the legislative debates (including protest dynamics, the “massacre law” label used by opponents, and the government’s safety framing) shows how conflict becomes organized around competing public goods—child safety, public order, animal welfare, and the legitimacy of municipal capacity claims—rather than around accurate assessments of what policies can realistically achieve [7,37]. Recent research on mediated publics in Turkey suggests that “street dogs as threats” functions as a recurring interpretive frame that is reproduced and disputed in public communication through moralized claims about safety and contested ideas of who can belong in shared urban space [38,39].

For caregivers, the amendment is experienced through everyday interfaces—hotlines, municipal routines, and shelter intake—where outcomes depend on discretionary decision-making and uneven capacity. Recent research on municipal dog management in Istanbul shows that it often proceeds through removal and displacement rather than stable welfare pathways [19,24,28,40,41]. Volunteers’ perspectives add a further layer to this existing scholarship: caregivers describe absorbing costs, logistical burdens, and conflict mediation in the gaps created by uneven municipal performance and limited infrastructure, particularly when institutional responses are delayed, opaque, or inconsistent [42]. In the post-amendment environment, institutional contact is therefore not simply a route to assistance but a decision with uncertain downstream effects that shapes when caregivers intervene and how visible they allow their caregiving to be.

This article draws on 43 in-depth interviews and five months of fieldwork conducted in Istanbul between July and November 2025. It reports by using coarse categories and avoids collecting routinized schedules, precise locations, or evasion tactics, treating restraint as an ethical requirement rather than methodological limitation. It combines constructivist grounded theory and reflexive thematic analysis to trace how governance is lived through the body, routine, and constrained pathways of assistance.

The findings are grouped into four interlocking themes: (1) anticipatory grief, chronic vigilance, and ambiguous loss as a temporality of “living in pre-loss” under institutional uncertainty; (2) shifting meanings of care and responsibility as commitment is narrowed into risk management; (3) encounters with municipal actors and shelters as discretionary, delay-prone, and opaque pathways that make help-seeking uncertain; and (4) veterinary bottlenecks and short-term holding gaps as recurrent points where care trajectories stall. The article traces how the 2024 amendment reshapes everyday caregiving through anticipatory loss, risk-managed responsibility, and gatekept institutional pathways, and shows how these dynamics produce time-sensitive delays with uneven welfare effects.

## 2. Materials and Methods

### 2.1. Research Design and Methodological Approach 

This research employed an interpretivist qualitative design to examine how recent legal and policy shifts have reshaped community caregiving for street dogs in Istanbul. The central analytic unit was the voluntary care situation, defined as the dynamic relationship among caregiver(s), particular dogs (or groups of dogs), and neighborhood context, as shaped by institutional responses (municipal services, shelters, complaint mechanisms) and veterinary access. This unit of analysis enables attention to mechanisms linking affect to welfare: how emotions such as chronic vigilance, anxiety, resentment, moral distress, and anticipatory grief are translated into changes in care capacity and, in turn, into everyday outcomes for dogs.

Conceptually, this study treats community caregiving as an informal but indispensable urban care infrastructure and approaches caregiver burden as relational and infrastructural—shaped by institutional uncertainty, neighborhood dynamics, and bottlenecks in veterinary access—rather than as an individual disposition.

Two complementary qualitative approaches were used: constructivist grounded theory (CGT) and reflexive thematic analysis (RTA). CGT guided inductive concept development grounded in participants’ terms and interpretive frameworks, enabling analytic sensitivity to local moral vocabularies and street-level understandings of legality. RTA was then used to develop analytically coherent, practice-relevant themes connecting affect, relationships, neighborhood dynamics, and institutional constraints. Themes were treated as interpretive outputs generated through iterative engagement with the dataset rather than as static “buckets” of codes.

This methodological pairing supported an animal-centered analytic trajectory: analysis moved from situated accounts (e.g., delayed intervention due to fear; stalled care due to lack of short-term holding; unpredictable hotline responses) to patterned configurations that matter for animal welfare—where and why care contracts or holds.

### 2.2. Participants, Sampling, and Interviews

To protect participants, I do not name districts or micro-localities. Place is treated as identifying, so I use four broad “urban fabric” categories: high-density inner-city mixed-use neighborhoods (*n* = 14), peri-urban expansion zones (*n* = 13), industrial and infrastructure corridors (*n* = 10), and low-density coastal/agricultural neighborhoods (*n* = 6). Years of involvement are reported in rounded terms.

Fieldwork was conducted in Istanbul between 1 July and 30 November 2025. Participants were adult community caregivers (≥18) providing unpaid street-dog care (feeding/monitoring, first aid and transport, ad hoc fostering, and informal mediation with neighbors and municipal actors). Inclusion required (i) ongoing caregiving, (ii) ≥12 months’ experience, and (iii) self-identification as an “animal lover.” Institutional actors (municipal teams, shelter staff) were not sampled.

Recruitment combined purposive and snowball sampling, seeded through multiple unconnected networks to reduce gatekeeper effects. The final sample comprised 43 caregivers with varied roles and experience. Interviews followed a do-no-harm protocol. Consent was obtained in writing when preferred and treated as ongoing; participants could skip questions or stop at any time. I explained the anonymization plan (coarse spatial categories, removal of micro-geographies, time-shifting, and composites where needed) and the limits of confidentiality, including procedures for imminent risk. This study also avoided collecting or retaining materials that could enable surveillance or punitive action (including municipal correspondence, complaint logs, and institutional case records) and relied on participant narratives and low-risk public observation reported in generalized form.

I conducted 43 semi-structured interviews in Turkish, usually in neutral venues chosen by participants and away from routine caregiving sites. Interviews were not audio-recorded; I relied on contemporaneous fieldnotes, and expanded notes written shortly afterward, with brief end-of-interview summaries to confirm accuracy and comfort. The guide emphasized experience and ethical reasoning rather than operational detail, covering changes since the legal amendment, affective impacts, institutional encounters, care infrastructures, and relations of attachment and loss. When operational specifics arose, discussion was redirected to structural or experiential aspects. Observation was limited to single-visit scans of public settings (parks, squares, marketplace edges). Notes focused on publicly visible signals, interactional cues, and access bottlenecks, and excluded clinic names, routes, plates, and precise timestamps. During heightened enforcement, in-person observation was paused and follow-ups were conducted remotely. Participants were assigned non-identifying codes at first contact. Materials were stored on encrypted drives, with consent records kept separately. Quotations were selected with a disclosure-risk screen: micro-sites, distinctive incidents, and identifiable third parties were removed; details were generalized; and high-risk passages were paraphrased or incorporated into composite vignettes.

### 2.3. Data Analysis

Interview notes and observational fieldnotes were compiled into a single analytic corpus and analyzed iteratively using a combined approach drawing on constructivist grounded theory (CGT) [43,44] and reflexive thematic analysis (RTA) [45,46]. Analysis proceeded in two coding cycles. First-cycle descriptive and NVivo coding prioritized participants’ own idioms, affective vocabularies, and locally salient legal and policy terms, preserving emic formulations and the texture of lived experience. Second-cycle pattern coding then clustered these codes into higher-order analytic categories and candidate themes linking affective states, everyday governance encounters, relational context, and care capacity (e.g., recurrent descriptions of “checking the phone at 3 a.m.,” coded as chronic vigilance; narrowed intervention as withdrawal; clinic species limits and boarding constraints as veterinary bottlenecks).

Following RTA principles, themes were treated as interpretive outputs developed through sustained engagement with the dataset rather than as fixed containers of codes. Theme development involved iterative drafting, revision, and refinement through analytic memoing and constant comparison across cases. Negative cases were actively sought to qualify and sharpen interpretations (e.g., accounts in which care capacity remained stable despite perceived threat; situations where neighborhood solidarity buffered distress). Where appropriate, interview accounts were situated through low-risk observation in public settings and triangulation across participants’ descriptions of institutional procedures and constraints, not to establish an external benchmark of “objectivity” but to locate interpretations within the everyday landscapes of care and constraint. This study did not incorporate municipal correspondence, hotline records, shelter files, or exchanges with municipal or shelter personnel as data sources.

To balance resonance with safety, limited participant feedback was sought only at the level of theme summaries, excluding quotations and timelines. Qualitative data management and coding were conducted in NVivo 12 Plus (version 12.6.1.466). Credibility was supported through method–theory fit (constructivist grounded theory and reflexive thematic analysis), iterative memo trails, attention to negative cases, and triangulation across interviews and low-risk observation. Transferability is intentionally bounded by Istanbul’s urban context and Turkey’s legal environment. Finally, the deliberate under-reporting of operational detail is treated as an ethical constraint rather than a methodological weakness, consistent with the risk of deductive disclosure in small-world networks; thick description is therefore provided at the level of care infrastructure (community networks, veterinary bottlenecks, relational dynamics) rather than traceable routines.

### 2.4. Institutional Review Board Statement

This study was conducted in accordance with the Declaration of Helsinki and approved by the Ethics Committee of Kadir Has University (protocol codes E-82741295-600-92377, approved on 14 July 2024, and E-82741295-600-123260, approved on 15 July 2025).

## 3. Results

Analysis of 43 in-depth interviews, supported by five months of qualitative fieldwork, identified four connected themes that describe how caregiving for free-living dogs in Istanbul has shifted under the uncertainty created by Turkey’s 2024 legal amendment. Participants did not treat emotion, policy, and infrastructure as separate domains; they described them as entangled in everyday practice—sleep disruption and constant checking, altered routines and visibility, and uneven or risky routes to help (municipal delays, opaque shelter intake, and recurring barriers to veterinary care).

Themes were developed by tracing recurring patterns across interviews, starting from participants’ own expressions and grouping them into shared processes. Presented separately for clarity, the themes overlap in practice: together they show how uncertainty reshapes what caregivers feel able to do, when they act, and how quickly dogs can receive care. The findings are organized into four themes: (1) chronic vigilance and “pre-loss”; (2) care narrowing into risk management; (3) discretionary and opaque municipal and shelter pathways; and (4) constrained veterinary care shaped by a short-term holding and recovery gap.

### 3.1. Theme 1: Anticipatory Grief, Chronic Vigilance, and Ambiguous Loss

Caregivers described the post-amendment period as living “on alert,” with disappearance or sudden municipal removal feeling possible at any moment. Many said this changed how they slept, how they paid attention, and how they moved through the day. Vigilance took two forms at once: constant monitoring for information (municipal updates, social media posts, neighborhood rumor) and a physical state—broken sleep, intrusive checking, and persistent arousal—that shaped where they went, when they went, and what they felt safe doing in public. In this climate, grief often arrived early. Caregivers spoke about staying attached to familiar dogs while also bracing—emotionally and practically—for loss that could come without warning and without confirmation, especially when dogs disappeared or were removed with no clear trace.

This anticipatory stance was repeatedly triggered by small, everyday cues. One caregiver explained that “the dogs disappearing for a few days triggers a panic that ruins my ability to focus on anything else” (Participant #9). Another described a nightly routine of checking hazards: “I check the perimeter fences twice a night… to ensure no dog got trapped or injured by the barbed wire” (Participant #10). Weather and infrastructure were treated as warning systems too: “When the forecast shows extreme cold, I spend the entire night trying to locate old blankets, unable to sleep” (Participant #9), and “My phone is permanently set to the weather app’s severe storm warnings. I can’t sleep until I’ve physically verified every shelter is dry and intact” (Participant #16).

Over time, this constant watchfulness left little room to recover. Participants described fatigue and thinning attention, which affected how consistently they could monitor dogs and how quickly they could respond when a dog was injured, missing, or exposed to sudden risks.

### 3.2. Theme 2: Shifting Meanings of Care and Responsibility—From Commitment to Risk Management

Across interviews, caregivers described a shift in what “responsible care” could realistically mean after the 2024 amendment. Many framed care less as an open-ended commitment and more as an ongoing assessment of risk: how visible they could be, what an intervention might trigger, and whether they could carry a case through to treatment and recovery. This was not described as caring less. It was described as caring under tighter constraints.

This shift showed up most clearly in everyday routines. Caregivers talked about care as constant coordination—feeding, checking dogs, arranging transport, managing minor injuries, and preventing crises—often carried by one person. Under pressure, that same work became more defensive. Participants described choosing times and routes more carefully, staying outside for shorter periods, and avoiding situations where they could not predict the consequences. Several said the question was no longer only “What does the dog need?” but also “Can I safely do this, and will it help—or make things worse?”

The emotional cost was especially visible in accounts of unfamiliar, newly abandoned, or injured dogs. Some participants described guilt, nausea, shame, or lingering distress after passing by a dog they could not take on. These were not one-time dilemmas but repeated moments that accumulated into moral distress—feeling forced to fall short of one’s own standards because capacity, safety, and follow-up care were uncertain. One caregiver put it plainly: “I still care deeply, but the care I can give is now limited… I have to think… at every step… if my intervention will help the dog or just put me at risk, and that question is killing me” (Participant #43). Another described the same recalibration as constant calculation: “It used to be about dogs. Now, every single move, every minute you spend outside, everything has consequences. You have to…” (Participant #43).

Caregivers also described keeping care going through informal workarounds. Some relied on small “under-the-table” budgets, informal credit, or quiet resource-sharing to cover food and veterinary bills. Others took on extra work or used creative income to sustain care: “I use the sale of their portraits to pay their bills” (Participant #27). At the same time, the ethics of mediated care appeared as a persistent ethical tension: “I film their suffering in exchange for donations, and I constantly grapple with the ethics of that exchange” (Participant #42). Documentation and social media appeared as double-edged: visibility could bring donations, transport help, or foster offers, but it could also feel ethically uncomfortable and, for some, risky.

Overall, participants described a narrowing of what care could look like—not because commitment disappeared, but because the conditions around them made expansive care harder to sustain.

### 3.3. Theme 3: Encounters with Municipal Actors and Shelters—Discretion, Delay, and Opaque Pathways

Caregivers described “the state” through everyday points of contact: municipal hotlines, field teams, shelter intake desks, and informal backchannels. What unsettled them was how much seemed to depend on discretion—who responded, how a case was classified, whether intake was accepted, and what (if anything) happened afterward. Many said outcomes were hard to anticipate and difficult to verify.

Because of this, institutional contact became part of the care calculus. Calling a hotline or requesting collection was not experienced as a straightforward route to assistance. It was described as a decision with uncertain downstream effects, including the possibility of removal without transparency, confinement under poor conditions, or outcomes caregivers could not track or contest. Several participants described hesitating to involve municipal services even in urgent cases because the “help” could also produce harm—or at least produce a chain of events they could not control.

Delays and unclear thresholds were common across accounts. Participants described unanswered calls, being redirected between units, shifting criteria for what counted as eligible, and inconsistent information across offices or teams. Time was repeatedly lost at the moments when time mattered most: injuries worsened, neighborhood tensions escalated, and early intervention windows closed. In this context, uncertainty was described less as a distant policy issue and more as an everyday condition shaping decisions—whether to escalate a case, how visible to be, and whether to rely on institutional pathways at all.

Over time, many caregivers said they learned an informal “map” of discretion—who was responsive, which channels were less risky, when to insist, and when to stay quiet. But this did not remove the underlying ambiguity. The same system that sometimes provided support also had the power to confiscate and confine. This made contact emotionally draining and high-stakes, and it fed back into other changes described in the results: increased vigilance, more cautious routines, and a stronger pull toward self-reliance and informal networks.

### 3.4. Theme 4: Veterinary Bottlenecks and Short-Term Holding Gaps

When municipal routes were experienced as slow, uncertain, or risky, caregivers often described private veterinary clinics as the fastest way to get a dog seen. Clinics were framed as one of the few places where treatment could begin immediately. But participants’ accounts also showed that “access” did not end at diagnosis or a procedure. It often broke down at the next step: where the dog could safely recover.

A recurring problem was short-term holding. Many clinics could treat dogs but could not keep them, even briefly, due to space limits, staffing, infection control concerns, or lack of kennels. Caregivers described cases that stalled over a narrow window (often 24–48 h): a dog needed warmth, monitoring, containment, and follow-up, but there was nowhere to bridge the time between street and recovery. In these moments, a treatable condition could escalate into a crisis—not because treatment was impossible, but because the recovery pathway was missing.

This gap pushed recovery work into precarious arrangements. Caregivers described scrambling for temporary fosters, negotiating with landlords, and turning bathrooms, balconies, or storage corners into makeshift recovery spaces. These arrangements carried costs: financial strain, conflict at home, eviction risk, and the fear that one more case would exceed what could be managed. This absence of holding space was often experienced as a daily logistical trap. Emergency transport itself is uncertain and requires risk management. One caregiver explained, “Finding someone with a car willing to navigate these blocks for an emergency transport is impossible. We are entirely isolated” (Participant #3). For some, the only mobility available was improvised and fragile. For others, transport was punishingly time-consuming: “I can’t use public transport; buses do not take street dogs… and I don’t have a car… which means I have to walk hours to the closest vet that offers a discount, which takes my entire day” (Participant #21).

For many caregivers, even routine feeding and monitoring involve physical hazards. As one participant explained, “I have to cross three lanes of heavy traffic to get to the main feeding spot while carrying boxes and bags of dog food, and sometimes bottles of water… I look homeless… and it is dangerous to cross the streets when no car stops for me” (Participant #26). As indicated in recent works on the access-to-care, transport and logistics are not secondary problems. Instead, they structure whether care happens at all and whether it happens in time, particularly for caregivers living in underserved communities [47,48,49,50,51,52].

When municipal pathways felt unreliable and clinics could not bridge holding gaps, caregivers described compensating through domestic and informal spaces, often at significant personal risk: “My house is a constant rotation of recovering dogs, and my landlord is threatening eviction. My sanctuary is my biggest risk” (Participant #19). Others built ad hoc infrastructures. For some caregivers, theft and sabotage further forced feelings of precarity and a tendency to further withdraw: “All my supplies are stolen or vandalized every two months. The only solution is to hide them… Then, what will I do in an emergency?” (Participant #30).

Over time, these bottlenecks reshaped caregiving capacities. Several participants described narrowing routes, avoiding situations they could not realistically carry through to recovery, and prioritizing dogs already embedded in known routines and support networks. Others described withdrawal as self-protection: “During feeding, there are some streets I intentionally refuse to go…” (Participant #2) and “I limit my route now…” (Participant #14). In participants’ accounts, the holding gap was not a minor inconvenience; it was a predictable failure point that redirected care into debt, secrecy, and triage—with direct consequences for dogs’ outcomes.

Together, these themes describe how care is narrowed by threat, opacity, and infrastructural bottlenecks, setting up the discussion of how welfare outcomes are shaped by constrained caregiving capacity.

## 4. Discussion

This article shows how Turkey’s 2024 amendment in Animal Protection Law is experienced by many street dog caregivers less as a distant legal change than as a set of everyday conditions that reshape what care can realistically look like. Caregivers described living with plausible loss, treating help-seeking as a calculated risk, and narrowing intervention to what they could sustain without triggering backlash or producing new harm. Across accounts, the most damaging effects were rarely a single removal or crisis; they accumulated through uncertainty—uneven municipal responses, opaque shelter pathways, and a recurring gap between clinical treatment and safe recovery. In this setting, caregiver well-being is not separable from animal welfare outcomes: it helps determine whether monitoring continues, whether intervention happens in time, and whether treatable cases move beyond emergency patchwork.

The discussion below situates these patterns in relation to existing work on care under threat, discretionary governance, and access to veterinary services, and then considers what kinds of bridge infrastructures could reduce caregiver strain while preventing predictable welfare breakdowns.

### 4.1. Anticipatory Grief as a Condition of Care

Participants described a present organized by sustained hypervigilance and anticipated loss, in which sudden removal, disappearance, or death of street dogs became an ordinary possibility rather than an exceptional rupture. These accounts align with work on continuous traumatic stress, which frames life under ongoing threat as a condition where vigilance is adaptive and time is oriented toward anticipated danger rather than post-event recovery [53,54]. They also echo research on animal-care labor documenting secondary traumatic stress, burnout, and compassion fatigue under high responsibility and limited organizational support [55,56,57]. Importantly, distress in this setting was not narrated as a private reaction to policy alone. It was tied to the social and media conditions in which care is visible and contested [29,38] and to the practical limits these conditions impose on routine, attention, and predictability.

Within this environment, grief was often described as anticipatory and unresolved. Anticipatory grief is typically discussed in relation to expected death, while ambiguous loss describes losses without closure—losses sustained by uncertainty and the inability to verify, register, or account for an absence [58,59,60]. Companion-animal bereavement research further notes that such grief is often disenfranchised, minimized, or treated as socially illegitimate, which can intensify isolation and compound distress [61,62,63,64]. In this study, “pre-loss” was tied less to illness trajectories than to disappearance, forced removal, and opaque institutional and medical processes. Analytically, this shifts anticipatory grief and ambiguous loss from a domestic end-of-life register to a governance register, where uncertainty is produced through institutional opacity, complaint-driven intervention, and discretionary enforcement.

These dynamics matter for welfare because they reorganize attention and monitoring in predictable ways. When caregivers reduce time in public, narrow their routes, or hesitate before escalating a case, minor problems can worsen, unfamiliar dogs are more likely to be missed, and early mediation of conflict becomes harder to sustain.

### 4.2. When Responsibility Becomes Risk Management

A central pattern across interviews was a shift in how “responsible care” was defined and practiced. Participants did not describe caring less; they described caring under conditions that made care risky. Commitment persisted, but it was repeatedly recalibrated into a situational ethics shaped by visibility risks, constrained support pathways, and the possibility that help-seeking itself could trigger removal or violence. This aligns with research showing that volunteer commitment can remain strong while being narrowed into risk management under constrained and complaint-sensitive environments [42]. It also fits scholarship on the moral organization of animal protection as a charged social field, where legitimacy, surveillance, and institutional scripts shape what “doing the right thing” can look like in practice [53,65,66].

This recalibration was most visible in routine. Outside institutional settings such as shelters, daily care was often described as improvised, frequently solitary coordination—feeding, checking on dogs, arranging transport, handling small injuries, and trying to prevent crises—what one body of scholarship characterizes as the grind of “keeping things going” [66]. In this framing, care appears less as a stable virtue than as a situated achievement: a negotiation of response-ability, understood as the practical capacity to respond to animals’ needs while remaining within shifting limits of time, safety, reputational exposure, and institutional volatility [67,68]. It also resonates with the argument that obligations are forged in entanglement rather than guaranteed in advance [69].

Ethnographic and organizational studies clarify why “commitment” becomes something to manage. Jun’s ethnography of a South Korean animal shelter shows how compassion becomes an organized labor regime—gendered, disciplined, and reputationally fragile—where commitment is continuously managed amid scrutiny [66]. Complementing this, institutional ethnographies of sheltering foreground how frontline care is coordinated through procedural texts, metrics, and cross-agency mandates (e.g., “One Welfare”), often leaving workers to improvise ethically and practically within gaps and accountability pressures [67,70,71,72]. Compassion can be drawn into moralizing regimes that discipline care, expose it to co-optation, and render commitment simultaneously demanded and precarious [66,67,70,73,74,75]. Research on “care-based animal dirty work” similarly emphasizes powerlessness, secrecy, stigma, and contested legitimacy as conditions that reorganize everyday ethics around keeping the work doable and socially survivable [55,74].

Participants’ accounts made the moral cost of this narrowing explicit. Several described the corrosive question that now shadows ordinary interventions: “I still care deeply, but the care I can give is now limited… I have to think… at every step… if my intervention will help the dog or just put me at risk, and that question is killing me” (Participant #40). Another described how consequence and calculation became ambient: “It used to be about dogs. Now, every single move, every minute you spend outside, everything has consequences. You have to calculate every action” (Participant #39). In these accounts, moral distress emerged less as one dramatic dilemma than as an accumulative experience of repeatedly falling short of one’s own standards under conditions that made expansive care difficult to sustain [48,57,76,77,78].

That recalibration also changed the boundaries of obligation. Encounters with unfamiliar dogs in need became especially fraught: “I saw a dog I didn’t know needing help, but I had to drive past. I felt sick. I am forced to limit my help to only the ones I know I can keep safe” (Participant #38). In practice, care concentrated around familiar dogs embedded in known routines and fragile protection networks, while newly abandoned, injured, or less familiar dogs were more likely to be deferred, passed by, or shifted into indirect support. A similar narrowing appears in institutional ethnographies of shelter work, where triage is shaped not only by scarcity but also by scrutiny, documentation requirements, and the need to produce defensible decisions under contested norms [70,79,80].

Care continued through informal workarounds and fragile economies. Participants described “shadow budgets,” informal credit, covert resource rerouting, and creative income streams to cover food and veterinary bills: “My stall profits used to determine my week; now, how much food I can buy for the sickest dog is my only metric for success” (Participant #1); “My clinical training focused on precision, but street care is just risk management, deciding which dog gets the one chance” (Participant #4); “the volume of protein scraps I can secretly divert” (Participant #8); and “client tips for food and medicine” (Participant #32). These patterns match ethnographic work on rescue economies where care depends on unstable finances and continuous moral accounting [81]. Participants also described the ambivalence of mediated care, where documentation can unlock resources while creating ethical discomfort: “My goal became documenting their life, not saving them… by raising awareness” (Participant #34); and “My care is inherently transactional. I film their suffering in exchange for donations, and I constantly grapple with the ethics of that exchange” (Participant #42).

### 4.3. Discretion and Opacity in Municipal and Shelter Pathways

Caregivers’ experiences of “the state” were most often routed through ordinary interfaces—hotlines, municipal field units, shelter intake desks, and informal backchannels—rather than through formal legal processes. What destabilized these encounters was the degree to which outcomes seemed to hinge on discretion: who answered the call, which unit arrived, how a case was classified, whether intake was accepted, and whether any follow-up could be verified. This aligns with classic accounts of street-level bureaucracy, where frontline actors effectively “make policy” through discretionary triage under constraint [80,81]. Qualitative research with voluntary caregivers in Istanbul similarly identifies municipalities as a major bottleneck in street-animal governance, including uneven fulfillment of responsibilities, inconsistent municipal action, and shelter inefficiencies that feed uncertainty about whether municipal involvement alleviates harm or intensifies it. Building on these dynamics, many critiques of the legal amendment argue that the prevailing “population management” repertoire of roundups, shelters, and removal from shared spaces redraws the boundary between “home” and “street,” recasting dogs in the commons as legitimate objects of containment and disposal [27,28,82,83,84]. In this frame, adoption can become a privatized transfer of care rather than a stable welfare solution, especially when trauma and its afterlives are managed by adopters with little public support [23].

Because municipal and shelter pathways were experienced as delay-prone and difficult to interpret, institutional contact often became part of the care calculus rather than a routine step in a care trajectory. Caregivers described learning an informal cartography of discretion—who was responsive, which channels were safer, when to insist, and when to stay quiet—while still feeling unable to predict consequences. Participants also described how these systems pull between “protection” and “control” mandates that do not sit comfortably together. In Srinivasan’s analysis of dog law and practice, governance operates through this dual aim, such that care is administered through the same institutional architectures that authorize seizure, confinement, and disposal; “help” becomes structurally difficult to distinguish from coercion in everyday encounters [85,86]. Other works also show how street dogs become governable through logics that legitimize exclusion and “eviction,” where state attention can signal heightened vulnerability rather than safety [87,88,89,90]. Institutional ethnography further explains why outcomes hinge on discretion as much as need: decisions are organized by legal thresholds, documentation demands, and procedural constraints that can delay or block intervention even when suffering is explicit [70,72,91]. These encounters also shaped expectations and alliances. Some participants reframed dogs within local ecologies of care and safety: “These dogs are our neighbors… they are also useful for the community…. Large dogs are making me feel safe; they keep the perimeter safe. The bond between me and them is so powerful. They keep me safe; I have to keep them safe” (Participant #20). Others described a shift in priority away from municipal framings of “public order” toward survival: “I was worried about aggression, but now my mission is less about public safety and purely about their survival” (Participant #10).

At the same time, the opacity of shelters and institutional processes produced distancing and withdrawal. One participant described risk-managed disengagement: “My contribution is purely financial leverage; I cannot risk physical interaction. This distance is both necessary and morally isolating” (Participant #30). Another captured the everyday moral trade-off under constraint: “Every fish scrap I give them is a calculated risk against my ability to pay my rent…I have to live through this risk. I don’t know if my efforts make any change, but it’s always a trade-off” (Participant #18). Others described the impossibility of the most time-sensitive cases within discretionary systems: “Nobody knows how to help or what to do to rescue dogs that are seriously injured, unfamiliar, newly abandoned” (Participant #15). Participants also noted how rationing becomes normalized: “the care I can give now is unavoidably rationed” (Participant #34), and “my help is limited only to dogs embedded in known routines and protection networks” (Participant #38).

In shelter work, emotionally difficult tasks—particularly intake and euthanasia—require continuous sensemaking to keep actions intelligible as caring even when painful, publicly contested, or procedurally constrained [92,93]. Recent works on shelter ethnography shows how legitimacy is stabilized through moral accounting and responsibility-shifting narratives that reorder guilt and sharpen judgments about “good” and “bad” care [94,95,96]. Volunteer-centered ethnography similarly shows how volunteers mobilize moral, relational, and reputational resources to contest routinized killing and to reframe “shelter death” as avoidable, making shelters a field of dispute over what responsible care should be [97]. Recent studies also highlight that care must pass through gatekeeping infrastructures—protocols, documentation requirements, decision rules—to become legible and authorized [79,80,98]. Where cases cannot be translated into recognizable categories of distress and the specific interventions those categories authorize, the result is often delay rather than care.

These delays have patterned welfare consequences. Unfamiliar dogs—often those displaced by municipal squads—typically arrive without stable caregivers, documentation, or medical records. When the window for effective treatment or safe de-escalation is narrow, even short delays can be consequential. Work on shelter systems suggests that late arrivals into strained infrastructures increase workloads and length-of-stay pressures, compromising rapid response and deepening welfare risk even when intake occurs [91,99,100,101].

### 4.4. The Street–Clinic–Recovery Gap and the Holding Bottleneck

Caregivers reported no reliable community veterinary service for street dogs—especially after hours—and no consistent access to admission-free emergency walk-in care. Municipally administered street-animal shelters are overpopulated and lack veterinary medical equipment and rehabilitation capacity [102]. When municipal response arrives late, or when the likely consequences of “calling for help” are unclear to the volunteers, care trajectories often shift toward private veterinary clinics as the most immediate and trusted points of intervention. This shift was not a simple preference for “better care” but a pragmatic adaptation to uncertainty. When caregivers could not predict whether municipal involvement would stabilize a case or intensify harm, they sought spaces where treatment could begin without procedural delay. This pivot aligns with broader research on access to veterinary care, which shows that delays emerge not only due to cost but also from the absence of reliable pathways, limited-service capacity, and the friction of navigating systems under time pressure [103,104,105,106,107,108,109].

Yet volunteers’ reliance on private veterinary services immediately produces another bottleneck: short-term holding. Caregivers repeatedly emphasized that many clinics may sometimes accept cats but cannot accommodate dogs even for brief stays because of space, staffing, and biosecurity constraints. The welfare crisis often hinged on a narrow temporal need, namely safe holding for 24–48 h, while caregivers stabilized a dog post-operation, arranged transport, negotiated foster space, or waited for a municipal decision that might never arrive. The gap was rarely clinical in a strict sense. It was infrastructural. A case could be treatable, even straightforward, and still collapse because there was nowhere for the dog to be safely kept once the clinic visit ended.

The shift to private clinics also expanded cycles of personal debt and informal credit. Care became increasingly tethered to fragile financial workarounds. As participants explained, “The informal credit system with the butcher shop is maxed out. Without that, the feeding operation stops” (Participant #8), and “The cost of a single vaccine is a week’s worth of my groceries. I can’t sustain this without external support” (Participant #16).

One participant stated bluntly, “I have to rely on over-the-counter (God knows may be smuggled) veterinary supplies because I cannot afford the official prices or the import duties” (Participant #42). Some managed the risks related to private veterinary services, debt, and unreliable medical treatments by remaining invisible: “I have to pay for everything in cash to avoid a paper trail that could expose me to my family and my job” (Participant #32). Even attempts to formalize asset protection collapsed under cost. As one caregiver explained, “I tried to set up a legal trust, but the lawyer demanded a fee I cannot pay. All my assets are personal and therefore at risk” (Participant #33).

This pattern closely matches research showing that financial barriers are the most frequently reported constraint to veterinary access and that such barriers reliably produce delayed or foregone care, particularly when animals are unowned and responsibility falls on under-resourced rescuers [57]. It also aligns with ethnographic work on animal rescue economies, where caregiving becomes inseparable from unstable accounting, credibility, and the ongoing effort to make resources appear where none formally exist [97].

As cases stalled, caregivers described a widening set of secondary labors, including searching for temporary space, coordinating transport, negotiating informal support, tracking recovery, and maintaining documentation under conditions that rendered “administrative competence” itself a scarce resource. As participants explained, “The data collection system I use is a series of hastily scribbled notes on napkins. I know the data is crucial, but I can’t find time to digitize it” (Participant #24), and “My documentation is on a single, old laptop that is constantly crashing. If I lose that, years of my work are gone” (Participant #34). Another caregiver lamented, “I was promised a space in the municipal shelter, but the paperwork is permanently stuck in a bureaucratic loop” (Participant #35).

These are the mechanisms through which care trajectories stall, not through a single failure but through accumulative friction across time-sensitive steps. The holding gap also intensifies moral distress and vigilance by forcing caregivers to bear the consequences of triage directly. Retreat and avoidance were often framed as self-protection rather than indifference. One participant explained, “During feeding, there are some streets I intentionally refuse to go and see the dogs because I know I cannot manage the hunger and the suffering of the packs there” (Participant #2).

Another caregiver described deliberate withdrawal from care and intentional self-restraint: “I limit my route now, deliberately avoiding the docks where I know the suffering is worse, to maintain my ability to function” (Participant #14). Some withdrew from direct intervention after repeated failures: “I used to try to catch them for neutering, but after failing three times and seeing their fear, I retreated to just feeding” (Participant #5).

Many others narrowed proximity to dogs. One participant explained, “I only feed them from a distance now; getting too close means realizing how much more I should be doing, which I can’t afford” (Participant #9). Others rationed time to preserve emotional capacity: “I only allow myself to spend a day with them. Any more and the emotional toll is too great to continue the next day” (Participant #20). These accounts align with the broader literature on compassion fatigue and secondary traumatic stress in animal care, where sustained exposure to suffering under constrained capacity predicts burnout, withdrawal, and emotional numbing [77].

In the most acute cases, the holding gap turned caregivers into reluctant arbiters of scarcity. As one participant described, “The ethical burden of deciding which dog gets the last dose of medicine is… sometimes…nauseating me… I just hate myself when I calculate which dogs should get the medicine… I avoid that decision as long as possible” (Participant #25). Another reflected, “My biggest struggle is… about…whether I am prolonging their suffering or genuinely helping them. It’s a constant moral tax” (Participant #31). Others described emotional hardening as a survival strategy: “I’ve become hardened to the deaths. I have to; otherwise, I would cease to continue my life… To be able to survive, I am trying not to feel or think about their dying” (Participant #16), or deliberate attachment: “I only focus on the ones I can save. I have created a psychological wall against the other 90%, and I have to live with that” (Participant #36). Some curtailed attachment altogether: “I have stopped naming them. I can’t afford the attachment; the grief is too much” (Participant #43). Others withdrew after failed attempts at private adoption: “I tried to adopt one but had to return it, and the shame of that failure has made me withdraw from all direct physical contact since” (Participant #39). Here, the clinic as a solution becomes ethically double-edged. It enables treatment, but without a holding bridge, transfers ongoing medical, logistical, and emotional responsibility back to individuals already operating at the edge of capacity.

Institutional threat compounds these bottlenecks. Many caregivers described environments in which reporting or insisting could jeopardize fragile access: “I constantly see signs of neglect and abuse from other caretakers, but I can’t intervene because I’ll lose my own precarious access to the area” (Participant #18). Another noted, “The police know who I am and what I do. I have to let some illegal acts of animal cruelty go unreported, just to maintain a fragile truce with the authorities” (Participant #41).

The bottleneck is therefore not limited to what a clinic can medically do. It is produced across the broader conditions that govern whether care can be carried out safely and continuously, including delayed municipal response, unclear handover procedures, the absence of lawful temporary spaces, and the risks attached to visible intervention. Within this landscape, short-term holding is pivotal rather than incidental. Evidence from shelter systems shows that even one or two nights in foster care can reduce stress and improve outcomes for dogs, demonstrating how brief periods of decompression and individualized attention can alter welfare trajectories. In street-dog cases, the same 24–48 h window often determines whether post-treatment recovery is secure, whether infection control is feasible, and whether a treatable condition deteriorates into irreversible harm. When municipal units arrive late and private clinics cannot hold dogs even briefly, this window becomes the point at which care most often breaks down, pushing cases into delay, worsening prognosis, and escalating costs borne privately by caregivers and bodily by dogs, consistent with veterinary access research linking barriers to delayed treatment and increased severity.

### 4.5. Limitations and Reflexive Considerations

I write from a dual position: scholar and long-term animal rights advocate involved in rescue and caregiving in Turkey. This position supported access, trust, and interpretive nuance, but it also raised familiar risks—confirmation bias, over-identification with caregivers’ accounts, and occasional role conflict—especially in advocacy settings marked by surveillance, stigma, and exposure concerns. I therefore treated ethics and positionality as ongoing, practice-based work, following an “ethically important moments” approach rather than relying on procedural approval alone [62].

Reflexivity was built into analysis through memo-writing across fieldwork and coding, bracketing assumptions before coding sessions, and peer debriefs with colleagues who were not involved in data collection. To reduce the risk of recognition of participants or sites, I deliberately generalized or “thinned” potentially identifying detail. This was an explicit ethical choice, consistent with the view that anonymization often requires compromise in dense networks and sensitive contexts [106,107,108]. These decisions were also guided by multispecies research ethics, which foreground responsibilities not only to human participants but also to animals and to the relational settings in which animal vulnerability is managed and politicized [108]. Finally, I attended to the affective and moral stakes of animal rescue and care which are associated with high levels of stress, secondary traumatic stress, and compassion fatigue. I treated the emotional texture of interviews as part of the research conditions, while remaining cautious not to let empathy substitute for analysis.

## 5. Conclusions

This study shows that the 2024 amendment reshapes volunteer caregiving through heightened threat, tighter scrutiny, uncertain institutional routes, and a recurring street–clinic–recovery bottleneck. Loss is often plausible but unconfirmable, sustaining chronic vigilance and shifting care from open-ended responsibility to managed exposure. Because municipal and shelter pathways are experienced as opaque, seeking help can feel like a gamble; many caregivers turn to private clinics for immediate treatment, only to encounter short-term holding gaps that derail recovery and push post-treatment care into homes and improvised spaces.

Withdrawal, then, should be read as a structured response to constraint rather than indifference. When cases must fit protocols to become actionable, delay and deferral become routine, and the work of managing time, risk, and fallout shifts back onto volunteers. The implication is straightforward: caregiver capacity is part of urban animal welfare. Reducing preventable suffering will require bridge infrastructures that make timely care feasible and low-risk—predictable municipal response, short-term holding and recovery options, and referral pathways that do not turn help-seeking into exposure.

## Data Availability

The data presented in this study are available on request from the corresponding author. The data are not publicly available due to privacy or ethical restrictions.
